# Soluble syndecan-1 and glycosaminoglycans in preeclamptic and normotensive pregnancies

**DOI:** 10.1038/s41598-021-82972-0

**Published:** 2021-02-23

**Authors:** H. Hassani Lahsinoui, F. Amraoui, L. J. A. Spijkers, G. J. M. Veenboer, S. L. M. Peters, N. van Vlies, L. Vogt, C. Ris-Stalpers, B. J. H. van den Born, G. B. Afink

**Affiliations:** 1 Department of Obstetrics and Gynaecology, Amsterdam University Medical Centers, University of Amsterdam, Meibergdreef 9, H4-222, 1105 AZ Amsterdam, The Netherlands; 2 Reproductive Biology Laboratory, Amsterdam University Medical Centers, University of Amsterdam, Amsterdam, The Netherlands; 3 Department of Vascular Medicine, Amsterdam University Medical Centers, University of Amsterdam, Amsterdam, The Netherlands; 4 Department of Internal Medicine, Spaarne Gasthuis, Haarlem, The Netherlands; 5 Laboratory Genetic Metabolic Diseases, Amsterdam University Medical Centers, University of Amsterdam, Amsterdam, The Netherlands

**Keywords:** Translational research, Animal disease models, Pre-eclampsia

## Abstract

Preeclampsia, an important cause of maternal and fetal morbidity and mortality, is associated with increased sFLT1 levels and with structural and functional damage to the glycocalyx contributing to endothelial dysfunction. We investigated glycocalyx components in relation to preeclampsia in human samples. While soluble syndecan-1 and heparan sulphate were similar in plasma of preeclamptic and normotensive pregnant women, dermatan sulphate was increased and keratan sulphate decreased in preeclamptic women. Dermatan sulphate was correlated with soluble syndecan-1, and inversely correlated with blood pressure and activated partial thromboplastin time. To determine if syndecan-1 was a prerequisite for the sFlt1 induced increase in blood pressure in mice we studied the effect of sFlt1 on blood pressure and vascular contractile responses in syndecan-1 deficient and wild type male mice. The classical sFlt1 induced rise in blood pressure was absent in syndecan-1 deficient mice indicating that syndecan-1 is a prerequisite for sFlt1 induced increase in blood pressure central to preeclampsia. The results show that an interplay between syndecan-1 and dermatan sulphate contributes to sFlt1 induced blood pressure elevation in pre-eclampsia.

## Introduction

The endothelial glycocalyx that covers the vascular wall is in direct contact with the blood flow. It consists of a complex network of membrane-bound proteoglycans and attached negatively-charged glycosaminoglycans (GAGs) that regulate various vascular functions such as permeability, leucocyte adhesion, coagulation and vascular tone by mediating shear-dependent nitric oxide (NO) release^1^ and facilitates VEGF signalling^2^.

Scavenging of vascular endothelial growth factor (VEGF) in the maternal circulation by its soluble receptor, vascular endothelial growth factor receptor 1 (sFLT1), contributes to endothelial dysfunction in preeclampsia^3,4^. Preeclampsia is defined by de novo hypertension and proteinuria during pregnancy and is an important cause of maternal and fetal morbidity and mortality^5^. Preeclampsia may trigger complex disorders in the endogenous coagulative pathways resulting in increased activated Partial Thromboplastin Time (aPTT)^6^.

In preeclampsia, excess sFLT1 is produced by the placenta, possibly in response to hypoxia that results from inadequate placentation^7,8^.

Syndecan-1 is the most abundant member of the syndecan family of proteoglycans that is also abundantly expressed in the syncytiotrophoblast layer in the chorionic villi of the human placenta^9,10^. The syndecan-1 signalling function is in part determined by the associated GAGs including heparan sulphate and dermatan sulphate^10^. Syndecan-1 regulates VEGF signalling by formation of a complex with the type 2 vascular endothelial growth factor receptor (VEGFR-2), thereby modulating VEGF-induced motility and migration of endothelial cells^11^. The binding of VEGF to VEGFR-2 is mediated by heparan sulphate that acts as a co-receptor^12,13^ and depletion of endothelial cell surface heparan sulphate results in reduced phosphorylation of VEGFR-2^14^. Heparan sulphate also possess a binding domain for sFLT1 serving as reservoir and limiting excess placental sFLT1 release into the circulation^15^.

Proteolytic shedding of transmembrane syndecan-1 yields (plasma) soluble syndecan-1, which retains its ability to interact with GAGs and growth factors in the circulation^16,17^. Placental *SDC1* expression has been shown to be diminished in preeclampsia^14,15,18,19^, and reduced soluble syndecan-1 at mid-pregnancy before the clinical onset of preeclampsia^20^. We hypothesize that the interaction between soluble syndecan-1 and its associated GAGs synergistically contributes to sFLT1-induced BP elevation characteristic of preeclampsia. In the present study, we assess whether circulating amounts of soluble syndecan-1 and associated GAGs are altered in preeclampsia compared to normotensive pregnancies and whether *Sdc1* deficient mice have a differential BP response to sFlt1 compared to wild type controls.

## Results

### Clinical characteristics of participants

A total of 125 pregnant women, 65 preeclamptic and 60 normotensive, were included. Clinical characteristics of pregnant women with and without preeclampsia are summarized in Table 1. HELLP syndrome (Hemolysis Elevated Liver enzymes and Low Platelets) was present in 18 (28%) women with preeclampsia. Magnesiumsulphate (MgSO_4_) was administered to 26 (40%) and anti-hypertensive treatment to 47 (72%) preeclamptic women. Of all women treated with antihypertensive medication, 40 (85%) were treated with a calcium-antagonist (nifedipine retard), 15 (32%) received a combined α1 and β-blocking agent (labetalol), 36 (77%) received a central α1 agonist (methyldopa) and 2 (4%) were treated with a selective serotonine 5-HT2-antagonist (ketanserin). Eleven (17%) women with preeclampsia were not treated with any type of antihypertensive medication, of these women, one received MgSO_4_ prior to delivery. There was no difference in gestational age at delivery between normotensive and preeclamptic women (239 ± 27 days vs 236 ± 27, *p* = 0.47). Part of the normotensive women were admitted for imminent spontaneous preterm labor. A calcium antagonist (nifedipine retard) was administered to 9 (15%) normotensive women as a tocolytic agent to delay imminent preterm labor.

**Table 1:** Clinical characteristics with comparison of normotensive pregnant women and women with pre-eclampsia.

Characteristics	Preeclamptic N = 65	Normotensive N = 60	*p*-value
Age, years	31 ± 5	29 ± 6	0.060
Caucasian*	27 (51%)	27 (60%)	0.370
Body Mass Index, kg/m^2^	27 ± 6	25 ± 7	0.100
Systolic BP, mmHg	154 ± 20	117 ± 14	< 0.010
Diastolic BP, mmHg	96 ± 11	68 ± 11	< 0.010
Proteinuria, g/24 h	1.50 [0.57–4.43]	N.D	–
Platelet count, × 10^9^/L	148 ± 84	234 ± 55	< 0.010
Lactate dehydrogenase	300 [239–551]	176 [136–213]	< 0.010
Nifedipine retard	27 (42%)	9 (15%)	0.000
Magnesiumsulphate	23 (35%)	0	0.000
Nulliparous^†^	35 (57%)	34 (58%)	0.980
Gestational age at delivery, weeks + days	33 + 5 ± 3 + 6	34 + 1 ± 3 + 6	0.470
Birth weight, grams	1914 ± 832	2292 ± 876	0.020
Birth weight percentile*	4.9 ± 1.6	6.2 ± 2.6	0.005
Soluble syndecan-1 (ng/ml)	553 [309–805]	550 [307–920]	0.957

Numbers represent mean ± standard deviation, median with [interquartile range], or number of subjects with percentages. ^†^Data missing for 4 women with pre-eclampsia and 1 normotensive woman. *Data on birth weight percentile missing for 18 women with pre-eclampsia and for 8 normotensive women. Between group differences were assessed by t-test for parametric, Mann–Whitney U test for non-parametric distributions and Chi square test for nominal variables. *p*-values were considered to indicate a significant difference if *p* < 0.05.

### Soluble syndecan-1 in preeclamptic and normotensive pregnancy compared to 3 months postpartum

Prior to delivery, soluble syndecan-1 concentrations in plasma of preeclamptic and normotensive women were similar. Levels were drastically decreased at 3 months postpartum in both groups (Table 2). Soluble syndecan-1 among normotensive pregnant women (551 ng/ml, IQR: 307–920), preeclamptic women with HELLP syndrome (644 ng/ml, IQR: 286–919 ng/ml) and preeclamptic women without HELLP (447 ng/ml, IQR: 267–734 ng/ml) are similar.

**Table 2:** Soluble syndecan-1 during normotensive and preeclamptic pregnancy compared to 3 months postpartum.

	Preeclamptic N = 4	Normotensive N = 5
Pregnancy	553 [309–805]	551 [307–920]
Postpartum	17 [15–18]	15 [10–17]

Numbers represent median and [interquartile range] in ng/ml. Postpartum soluble syndecan-1 was determined in 5 previously normotensive pregnant women and 4 previously preeclamptic women.

### Glycosaminoglycans in preeclamptic and normotensive pregnancy and at 3 months postpartum

**Figure 1:**
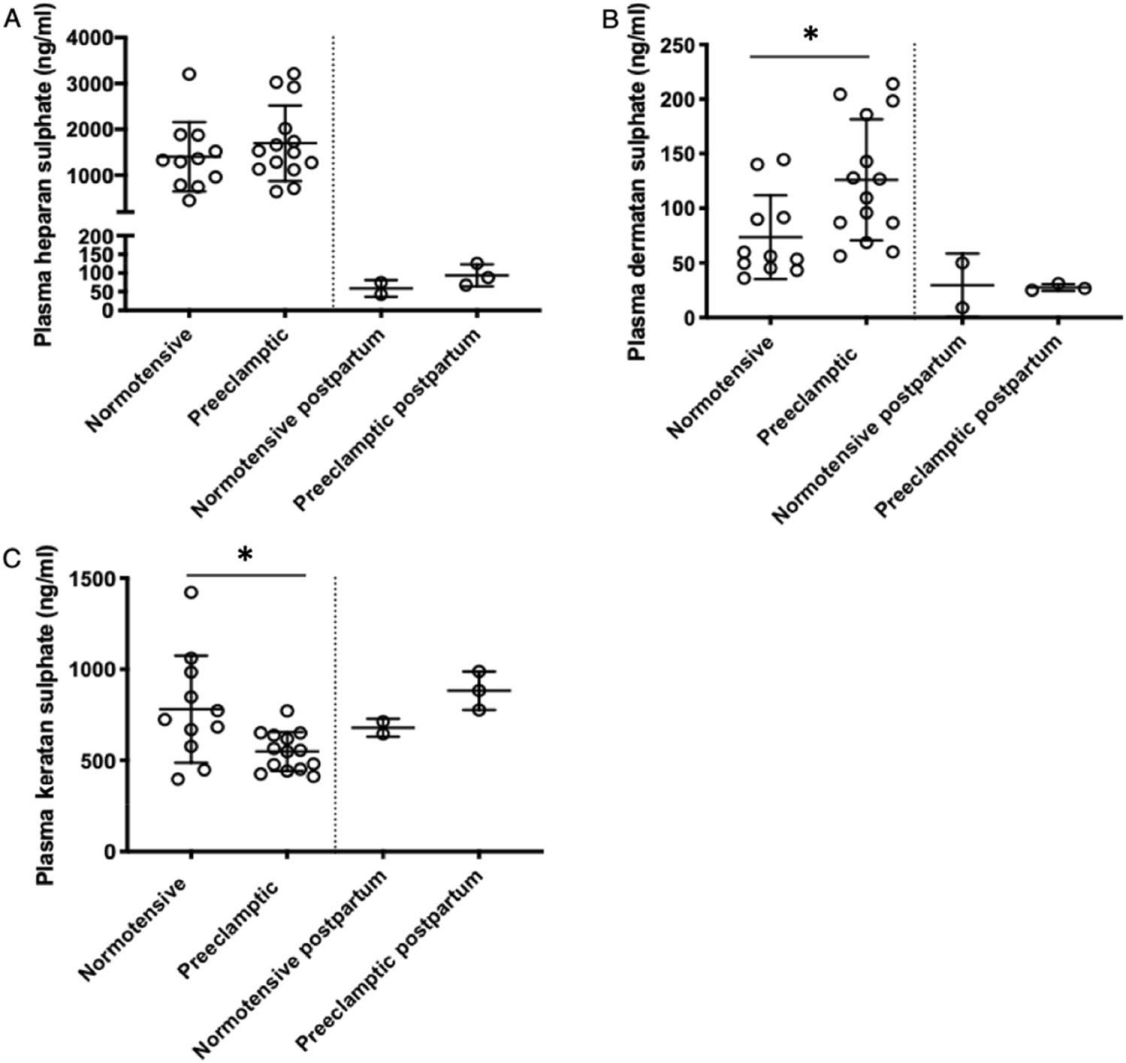
Plasma glycosaminoglycans during pregnancy and 3 months postpartum. Plasma glycosaminoglycans measured using HPLC–MS/MS. Bars represent mean with standard deviation. Plasma heparan sulphate in (**A**), dermatan sulphate in (**B**) and keratan sulphate in (**C**) are compared among normotensive pregnant women (NT, N = 11) and preeclamptic women (PE, N = 14) prior to delivery. Postpartum values were measured in 3 preeclamptic women and 2 normotensive women from the same population.* Indicates *p* < 0.01.

Plasma heparan sulphate concentrations in women with preeclampsia were similar to normotensive women prior to delivery (N = 14–11, *p* = 0.36) and significantly decreased at 3 months postpartum, (Fig. 1A). Plasma dermatan sulphate concentrations were significantly higher in preeclamptic women compared to normotensive pregnant women (N = 14–11, *p* = 0.01, Fig. 1B). In both groups, dermatan sulphate levels decreased to a similar extent 3 months postpartum (N = 3–2). In contrast, plasma keratan sulphate was decreased in preeclamptic women compared to normotensive controls (N = 14–11, *p* = 0.01) prior to delivery with comparable levels postpartum (N = 3–2, Fig. 1C).

### Soluble syndecan-1 is inversely correlated with blood pressure in preeclamptic women

**Figure 2:**
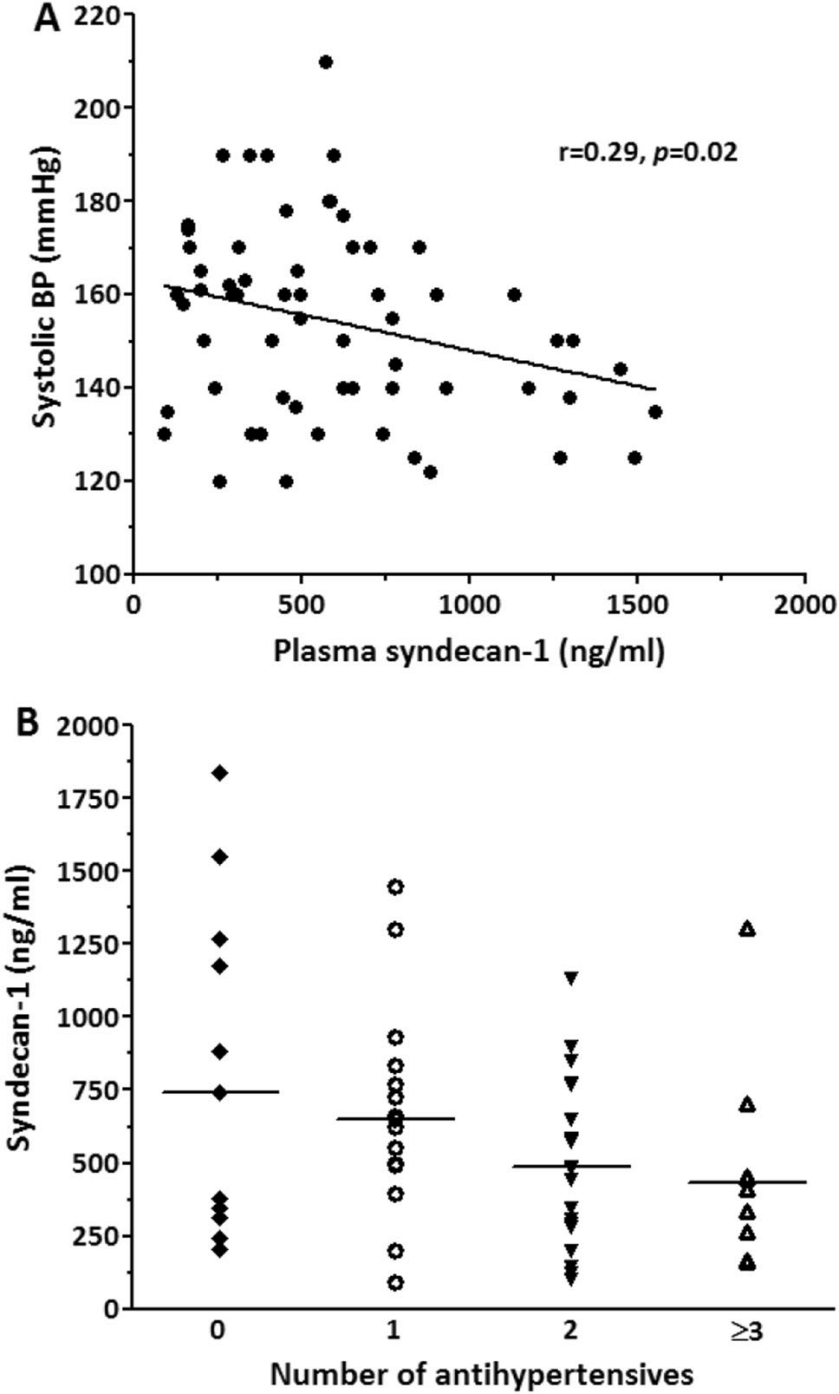
Soluble syndecan-1 levels in plasma are correlated with blood pressure and antihypertensive medication in women with preeclampsia. Correlation of soluble syndecan-1 with systolic blood pressure showing increasing blood pressure values with lower soluble syndecan-1, N = 59 (**A**). Soluble syndecan-1 among preeclamptic women categorized by number of received antihypertensive medication, from left to right: N = 11, N = 13, N = 16 and N = 7 (**B**). Median soluble syndecan-1 tends to decrease with increasing number of antihypertensives, but this was not statistically significant. Each symbol represents an individual patient with preeclampsia, the horizontal line represents the median.

In preeclamptic women, soluble syndecan-1 was inversely correlated with BP showing higher BP values with decreasing plasma concentrations of soluble syndecan-1 (Fig. 2A). This association was also evident from the number of required antihypertensive medication, which tends to be lower in women with higher soluble syndecan-1 values (Fig. 2B). This trend however, was not statistically significant. In normotensive pregnant women, soluble syndecan-1 was not correlated with systolic BP (r = 0.05, *p* = 0.75).

### Dermatan sulphate is strongly correlated with soluble Syndecan-1 and blood pressure in preeclamptic women

**Figure 3:**
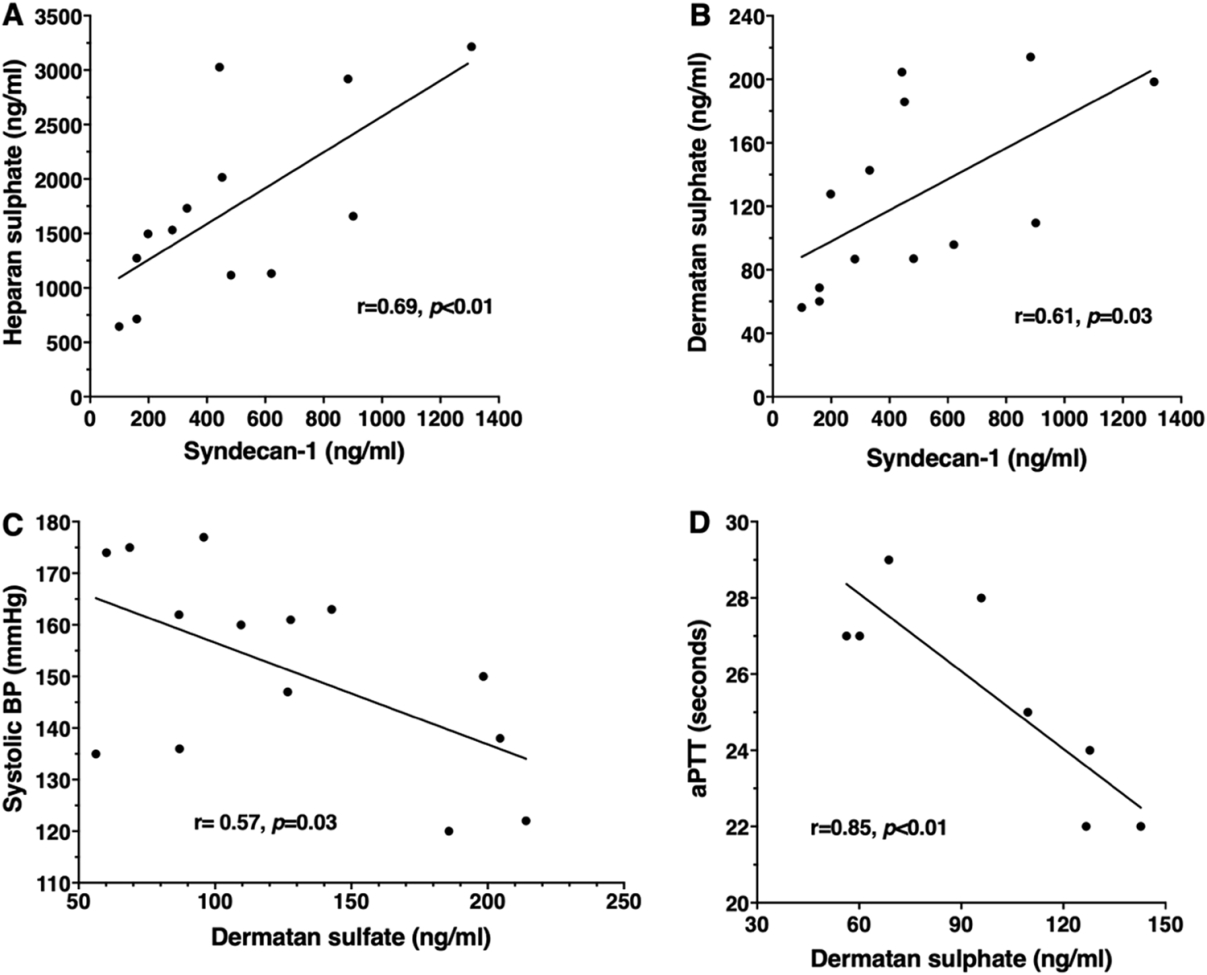
Association of plasma glycosaminoglycans with soluble syndecan-1, blood pressure and aPTT in women with preeclampsia. Correlation of plasma heparan sulphate (**A**) and dermatan sulphate (**B**) with soluble syndecan-1 in preeclampsia. Dermatan is inversely correlated with the highest systolic blood pressure (**C**) and activated partial thromboplastin time (aPTT) in women preeclampsia (**D**). Each dot represents an individual patient (**A** N = 13, B N = 13, C N = 14, D N = 8). Correlations are absent in normotensives.

Plasma heparan sulphate and dermatan sulphate were strongly correlated with soluble syndecan-1 in women with preeclampsia (Fig. 3A,B). In contrast, dermatan sulphate was inversely correlated with systolic blood pressure in women with preeclampsia compared to normotensive women (Fig. 3C), but not in normotensive pregnant women (r = 0.46, *p* = 0.25). Interestingly, the relatively low dermatan sulphate levels associated with increased systolic blood pressure also correlated with high activated Partial Thromboplastin Time (aPTT) (Fig. 3D).

### sFlt1 caused blood pressure (BP) elevation in wild type but not in syndecan-1 deficient mice

**Figure 4:**
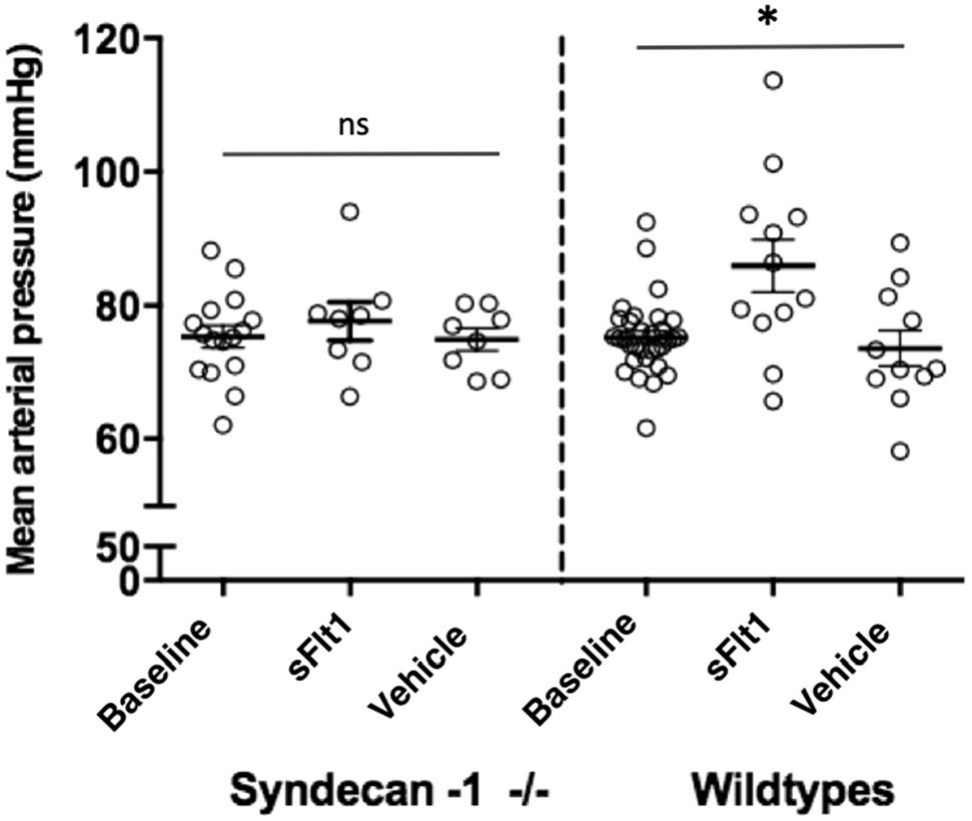
Effect of sFlt1 and vehicle on blood pressure of syndecan-1 deficient mice and wild type controls. Effect of sFlt1 or vehicle infusion during two weeks on mean arterial blood pressure (MAP) of syndecan-1 deficient mice and wild type controls. Syndecan-1 deficient mice have similar MAP at baseline (N = 16) and after treatment with sFlt1 (N = 8) and vehicle (n = 8). Treatment with sFlt1 significantly elevates MAP in wild type control mice (N = 12) compared to baseline (N = 32) and vehicle (N = 11) treatment. Bars represent mean ± SEM, ns indicates not-significant, * indicates *p* < 0.01.

To assess whether syndecan-1 contributes to sFlt1-induced BP elevation, we treated syndecan-1 deficient mice and wild type controls with sFlt1 or vehicle (PBS) for a period of two weeks. Baseline mean arterial blood pressure (MAP) in syndecan-1 deficient (75 ± 2 mmHg,) and wild type mice (75 ± 1 mmHg, N = 16–32, *p* = 0.95) was similar. In wild type mice, sFlt1 infusion induced a MAP elevation to 86 ± 4 mmHg, N = 12, *p* < 0.01), while MAP remained unchanged after vehicle-treatment (74 + 3 mmHg, N = 11, *p* = 0.55). In contrast, MAP of syndecan-1 deficient mice, remained unchanged after sFlt1 (78 ± 3, N = 8) or vehicle (75 ± 2 mmHg, N = 8) treatment (*p* = 0.66, Fig. 4).

### Isometric tension measurements (wire myography) in isolated carotid arteries from syndecan-1 deficient and wild type mice

**Figure 5:**
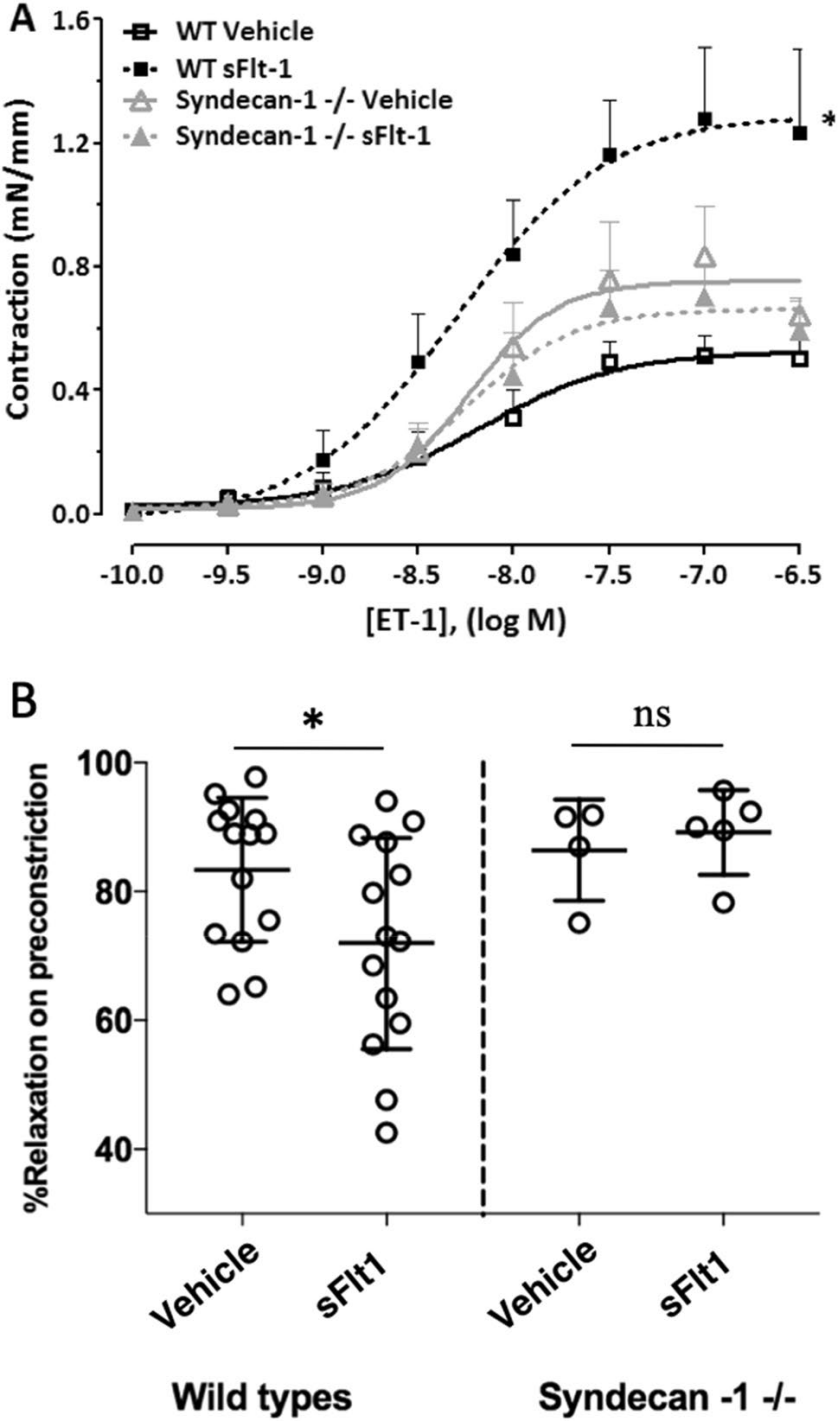
Endothelin-1-induced contraction in sFlt1 and vehicle-treated on syndecan-1 deficient mice and wild type controls. Syndecan-1 deficient mice and wild type controls were treated with sFlt1 or vehicle during two weeks. Carotid arteries were isolated for comparison of contractile responses, vasoconstriction is expressed in milliNewton per millimeter (mN/mm). (**A**) In wild types (WT), sFlt1 infusion augments maximal endothelin-1-induced vasoconstriction compared to vehicle (N = 6 vs. 6, *p* = 0.03). In sydencan-1 -/- mice, endothelin-1-induces vasoconstriction similar after sFlt1 and vehicle treatment (N = 5–4, *p* = 0.68). (**B**) In wild types, maximal methacholine-induced vasodilatation (after preconstriction with phenylephrine) is decreased in sFlt1-treated compared to vehicle-treated mice (N = 14 vs 14, *p* = 0.04). In sydecan-1 -/- mice, maximal methacholine-induced vasodilatation is similar after sFlt1 and vehicle treatment (N = 5–4). Bars represent mean ± SEM, ns indicates not-significant.

In isolated carotid artery segments of wild type mice, maximal endothelin-1-induced vasoconstriction after sFlt1 treatment (1.2 ± 0.3 mN, N = 6) was increased compared to vehicle treatment (0.5 ± 0.1 mN, N = 6, *p* = 0.03), while in syndecan-1 deficient mice, endothelin-1-induced vasoconstriction was similar after sFlt1 (0.59 ± 0.10 mN, N = 5) and vehicle treatment (0.64 ± 0.06 mN, N = 4, *p* = 0.68, Fig. 5A). In wild types, maximal methacholine-induced vasodilatation (after preconstriction with phenylephrine) decreased after sFlt1 treatment (72 ± 4%, N = 14), compared to vehicle treatment (83 ± 3%, N = 14, *p* = 0.04). In syndecan-1 deficient mice, maximal methacholine-induced vasodilatation after sFlt1 treatment (89 ± 3%, N = 5) and vehicle treatment was similar (86 ± 4%, N = 4, *p* = 0.58, Fig. 5B).

## Discussion

In the present study, we show that in contrast to normotensive pregnant controls, circulating dermatan sulphate is significantly increased in pre-eclampsia and strongly correlates with soluble syndecan-1 in preeclamptic women. In preeclampsia, both soluble syndecan-1 and dermatan sulphate are inversely correlated with blood pressure (BP). In addition, sFlt1 induces BP elevation in wild type mice, but not in syndecan-1 deficient mice, indicating that syndecan-1 may be an important mediator in sFLT1 induced blood pressure elevation in preeclampsia and anti-angiogenic hypertension.

The circulating soluble syndecan-1 levels we measure are similar to those reported previously^19,21^, but in contrast to previous studies, we dit not observe any difference compared to gestationaly aged matched normotensive controls^22^. This discrepancy may be explained by increased gestational age of controls in the previous studies compared to ours^19,21^ or by the high incidence of low birthweight (under 10th percentile) in our control group. Kaitu'u-Lino et al., has shown that in pregnancies resulting in the birth of a baby small for gestational age, syndecan-1 levels are significantly lower compared to controls^23^. The gestational age of normotensive controls in the current study was matched to that of the preeclamptic group by including women with preterm labor of unknown cause. Gandley et al. showed that soluble syndecan-1 increases with gestational age, possibly explaining the lower soluble syndecan-1 among normotensive controls in the present study.

Our pilot data confirms the massive decrease of soluble syndecan-1 after delivery in both normotensive and preeclamptic pregnancies reported previously^21^. Due to the small sample size this outcome needs to be confirmed in a larger patient cohort. This finding suggests that in pregnant women soluble syndecan-1 mainly originates from the placenta. *SDC1* is expressed on syncytiotrophoblast cells, which are in direct contact with maternal blood^21^. Shedding of syndecan-1 from the syncytiotrophoblast would likely increase soluble syndecan-1 in maternal plasma during pregnancy. Placental expression of *SDC1* has been shown to be reduced in preeclampsia by multiple independent studies^14,15,18,19^, possibly explaining the reduced plasma soluble syndecan-1 at mid-pregnancy reported previously^21^. In the present study, we corroborate the observation by Gandley et al. that soluble syndecan-1 is correlated with BP in women with pre-eclampsia^21^. Soluble syndecan-1 was not significantly different in preeclamptic and normotensive pregnancies.

In our experiments male mice were used. The main focus of the mice experiments was to gain more insight in the underlying mechanism by which soluble syndecan-1, GAGs and sFLT-1 alter blood pressure irrespective of pregnancy. The use of male mice could be a limitation in our study. However, a randomized controlled trial comparing placebo to monoclonal antibodies to VEGF in (mostly male) patients with metastatic clear-cell renal carcinoma resulted in a significant increase in the occurrence of hypertension and proteinuria in the treatment group^24^. Therapies that target VEGF and its receptors are known to induce “ preeclampsia like syndrome” (hypertension and proteinuria)^25^. These side effects occur in both females and males. The same phenomenon can be observed in mice^26,27^. This suggest a comparable underlying mechanism for anti-VEGF induced hypertension and proteinuria in both males and females.

In a *Sdc1* knockout mouse model, infusion of sFlt1 did not induce BP elevation. We previously showed that increased endothelin-1-mediated vasoconstriction and decreased methacholine-induced vasodilatation contributed to sFlt1-induced BP elevation in wild type mice^26^. These ex vivo effects on contractility were absent in syndecan-1 deficient mice in our study, suggesting that syndecan-1 is essential for sFLT1-induced hypertension. The potential effect of soluble syndecan-1 on BP may be mediated through its attached glycosaminoglycans, as the structure of these attached glycosaminoglycans are generally not affected in syndecan-1 deficient mice^28^. Administration of sulodexide, a mixture of GAGs containing 20% dermatan sulphate, significantly lowers BP in hypertensive and normotensive individuals^29^, possibly by mediating binding of circulating VEGF to the endothelium^30^. This hypothesis is supported by the positive correlation of soluble syndecan-1 with heparan sulphate and dermatan sulphate and the strong inverse correlation of dermatan sulphate with BP in preeclamptic women in this study. The blood pressure used in our analysis was the highest measured blood pressure during admission. The blood sample was taken at an independent time point during admission. The effect of this difference in sampling on the correlation is difficult to predict. Most of our patients with preeclampsia used one or more types of blood pressure medication. We must therefore take into account the potential role of blood pressure medication on syndecan-1 and blood pressure levels.

Like soluble syndecan-1, heparan sulphate is drastically increased in both preeclamptic and normotensive pregnancy and much lower postpartum, suggesting that heparan sulphate predominantly originates from the placenta. However, the syncytiotrophoblast, which is in direct contact with maternal blood has been shown to be devoid of dermatan sulphate^31^, providing support to a maternal endothelium origin of excess dermatan sulphate. In addition, endocan, an endothelial-cell specific soluble dermatan sulphate proteoglycan, is elevated in women with preeclampsia and positively correlates with circulating sFLT1^32^. One could hypothesize a potential protective effect by the increase of dermatan sulphate originating from maternal endothelium resulting in counteracting the increase in blood pressure seen in preeclampsia. In addition to interaction with sFLT1, dermatan sulphate is able to bind Transforming Growth Factor-β (TGF-β)^33^. Disruption of TGF-β signaling has been linked to endothelial dysfunction and decreased nitric-oxide availability in preeclampsia^3^. TGF-β signaling is modulated by soluble endoglin, which is also positively correlated with the soluble dermatan sulphate proteoglycan endocan in preeclampsia. Although merely speculative, elevated circulating dermatan sulphate levels may also be related to BP via interaction with TGF-β. Further support for a functional role of elevated dermatan sulphate in the circulation of preeclamptic women is provided by our observation that dermatan sulphate is strongly correlated with activated Partial Thromboplastin Time (aPTT) that reflects the integrity of the intrinsic clotting cascade known to be disrupted in preeclampsia^6^. The increase in aPTT has been associated with severe preeclampsia and the inverse correlation with dermatan sulphate may suggest a protective effect of dermatan sulphate in preeclampsia^6^. Accordingly, dermatan sulphate has previously been identified as a major determinant of coagulation in the placenta via activation of the thrombin inhibitor heparin cofactor II within fetal vessel walls^31^.

In conclusion, dermatan sulphate is significantly elevated in preeclampsia and inversely correlated with soluble syndecan-1 and BP, suggesting that the association of soluble syndecan-1 with BP in preeclampsia might be mediated by dermatan sulphate. Prospective studies are needed to assess whether administration of dermatan sulphate reduces the risk of preeclampsia or limits its severity.

## Materials and methods

### Study population

Clinical data and plasma samples were obtained from participants admitted to the department of obstetrics of the Amsterdam Medical Centre, The Netherlands after obtaining informed consent. Blood samples were drawn from participating pregnant women admitted to our obstetric ward. The participating women were not in active labor at the time of sample collection. Samples that had sufficient residual plasma left were selected for analysis of circulating GAGs. The study has been performed in accordance with the Decleration of Helsinki and was approved by the Medical Review Ethics Committee of the Amsterdam University Medical Centre, The Netherlands. Plasma was immediately stored at − 80 °C. Blood was also drawn at 3 months postpartum from the same study population. Preeclampsia was defined by systolic BP ≥ 140 mmHg or diastolic BP ≥ 90 mmHg recorded on two occasions at least 4 h apart, after 20 weeks gestation in a previously normotensive woman combined with new-onset proteinuria with urinary protein excretion ≥ 300 mg/24-h. BP was measured manually in the sitting position at the right upper arm using an aneroid sphygmomanometer. Diastolic BP was determined at Korotkoff sound V. For this study, the highest measured blood pressure during admission was included. Birth weight percentiles were assessed according to the local Dutch birth weight percentiles. The appropriate chart was chosen based on parity and gender of the baby (http://www.perinatreg.nl/). Small for gestational age was defined as birth weight below 10th percentile. HELLP syndrome was defined by lactate dehydrogenase ≥ 600 U/L or haptoglobin < 0.2 g/L, aspartate or alanine aminotransferase ≥ 70 U/L, and platelet count < 100 * 10^9^/L. Experiments were carried out in accordance with the declaration of Helsinki after informed consent from the participants was obtained. Experiments were approved by an independent ethics committee^4,34,35^.

### Soluble syndecan-1 and glycosaminoglycan analysis

Maternal plasma soluble syndecan-1 concentrations were measured with a commercially available human syndecan-1 enzyme-linked immunosorbent assay (CD138 ELISA Kit, Diaclone), according to the manufacturers’ instructions. Plasma GAGs (heparan sulphate, dermatan sulphate and keratan sulphate) were measured in a subset of participants (that had sufficient material for analysis left) using high performance liquid chromatography tandem mass spectrometry (HPLC–MS/MS) as previously described in detail^36,37^. heparan sulphate, dermatan sulphate, and keratan sulphate were first enzymatically digested into disaccharide units and then quantified on a Waters Quattro Premier XE (tandem) mass spectrometer (Waters Corporation, Milford, MA, USA) coupled to an Acquity UPLC system (UPLC-MS/MS). According to the nomenclature by Lawrence et al.^37^, heparan sulphate was represented by the sum of disaccharides D0A0, D0S0, D0A6 and D2A0, D0S6 and D2S0, dermatan sulphate was represented by D0a4 and D0a10 and keratan sulphate was represented by the sum of disaccharides G0A6 and G6A6. All samples were analyzed in triplicate^34^.

### Effect of sFlt1 on blood pressure in wild type and syndecan-1 deficient mice

To investigate the role of syndecan-1 signalling in sFlt1-induced BP elevation, we compared in vivo BP response and isometric tension measurements ex vivo in isolated arteries of syndecan-1 deficient mice and wild type controls. All experimental procedures including housing conditions, BP measurement and isometric tension measurements have been described in detail previously^26^. Briefly, adult (12–14 weeks old) male C57/BL6N mice (Charles River, Maastricht, The Netherlands) and *Sdc1* -/- mice on a C57/BL6N background were individually housed in a temperature controlled room with a 12:12 light–dark cycle with food and water ad libitum. After acclimatisation, mice were anesthetized for implantation of osmotic minipumps (Alzet, California USA). Pumps were filled with either vehicle (PBS) or sFlt1 (Creative Biomart, New York, USA, Catalog no: Flt1-1785 M) for continuous 0.5 µl/h compound release (equals 500 ng/h sFlt1) during 2 weeks. During treatment, BP was recorded at fixed time points using the non-invasive tail cuff BP measurement system, according to the previously described protocol (CODA system Kent Scientific Corporation, CT, USA). To reduce the adverse stress response to BP measurements, animals were trained during one week prior to the experiment. After 2 weeks, mice were euthanized and carotid arteries were isolated for isometric analysis of vasomotor tone. All experimental procedures were performed in compliance with the ARRIVE guidelines and approved by the Animal Ethics Committee of the Amsterdam University Medical Centre, The Netherlands^34^.

### Wire-myograph analyses

Carotid arteries were isolated and immediately placed in Krebs–Henseleit buffer (pH 7.4; in mM: 118.5 NaCl, 4.7 KCl, 25.0 NaHCO_3_, 1.2 MgSO_4_, 1.8 CaCl_2_, 1.1 KH_2_PO_4_ and 5.6 glucose) for connective tissue removal. Subsequently, one 2 mm (one segment per animal) artery segment per animal was mounted into a multichannel wire myograph for isometric tension measurements. Vessel segments were incubated with carbogen (95% O_2_, 5% CO_2_) aerated Krebs–Henseleit buffer at a temperature of 37 °C. During the experiment buffer was replaced every 15 min. In all experiments, the segments were first contracted with high K^+^-containing Krebs buffer (pH 7.4; in mM: 23.2 NaCl, 100 KCl, 25 NaHCO_3_, 1.2 MgSO_4_, 1.8 CaCl_2_, 1.1 KH_2_PO_4_ and 5.6 glucose). After 30 min washout, a concentration–response-curve (CRC) of the α1-adrenoceptor agonist phenylephrine was generated in half-log concentration increments (1 nM–0.1 µM). Contraction on phenylephrine was immediately followed by a methacholine CRC (1 nM- 1 µM) to assess endothelium-dependent vasodilatation. Next, after 15 min of washout a high K^+^ Krebs-induced CRC (5 mM–100 mM) was generated. Finally, an ET-1 CRC (0.1 nM–0.3 µM) was generated in half-log concentration increments. Contractile force of carotid artery segments is expressed in milliNewton per millimeter (mN/ml)^26,34^.

### Statistical analysis

Continuous clinical variables are expressed as mean ± standard deviation (SD) or median and interquartile range (IQR) for variables with a skewed distribution. Categorical data are expressed as number and percentages. Between group differences were assessed by t-test for parametric and Mann–Whitney U test for non-parametric distributions. One-way ANOVA or Kruskall-Wallis were used for comparison of more than two groups for parametric and non-parametric distributions respectively. Chi-square statistics were used for categorical variables. Linear regression analysis was used to assess the correlation of soluble syndecan-1 and GAGs with BP. For animal experiments, BP and isometric tension measurements are presented as means ± standard error of the mean (SEM). Independent *t*-test was used to compare wild type and syndecan-1 deficient mice. Statistical analyses were performed using Graphpad Prism software and SPSS (Statistical Package for the Social Sciences, version 23.0, Inc. Chicago, Illinois, USA). *p*-values were considered to indicate a significant difference if *p* < 0.05^34^.

## Supplementary Information

Supplementary Information.
