# Supplementary material for: Soluble syndecan-1 and glycosaminoglycans in preeclamptic and normotensive pregnancies
Source: Sci Rep. 2021 Feb 23;11:4387. doi: 10.1038/s41598-021-82972-0 (PMC7902809; doi:10.1038/s41598-021-82972-0)
Supplement: Supplementary file 1 — Supplementary Information. [file 41598_2021_82972_MOESM1_ESM.docx]

**Supplementary Data**

**Soluble Syndecan-1 and Glycosaminoglycans in Preeclamptic and Normotensive Pregnancies**

H. Hassani Lahsinoui**^1,2*^**, F. Amraoui**^3*^,** L.J.A. Spijkers**^3^**, G.J.M. Veenboer^2^**,** S.L.M. Peters**^3^**, N. van Vlies**^5^**, L. Vogt**^3^** , C. Ris-Stalpers**^1,2^**, B.J.H. van den Born**^3^** , G.B. Afink**^2^**

**Running Head: Role of soluble Syndecan-1 in Preeclampsia**

1. Department of Obstetrics and Gynaecology, Amsterdam University Medical Centers, University of Amsterdam, The Netherlands

2. Reproductive Biology Laboratory, Amsterdam University Medical Centers, University of Amsterdam, The Netherlands

3. Department of Vascular Medicine, Amsterdam University Medical Centers, University of Amsterdam, The Netherlands.

4. Department of internal medicine. Spaarne gasthuis. Haarlem, The Netherlands.

5. Laboratory Genetic Metabolic Diseases, Amsterdam University Medical Centers, University of Amsterdam, The Netherlands

* Both authors contributed equally

^§^Corresponding author. *Correspondence to:* H. Hassani Lahsinoui, Department of Obstetrics and Gynaecology, Amsterdam University Medical Centers, University of Amsterdam, Meibergdreef 9, H4-222, 1105 AZ, Amsterdam, The Netherlands. Tel. +31205665915, e-mail: [h.hassanilahsinoui@amsterdamumc.nl](mailto:h.hassanilahsinoui@amsterdamumc.nl)

**Supplementary table 1**

**Supplementary data**

**Table 1. Clinical characteristics with comparison of normotensive pregnant women and women with pre-eclampsia included in plasma glycosaminoglycans (GAGs) analysis.**

| **Characteristics** | **Preeclamptic**  **N=20** | **Normotensive**  **N=14** | ***P*-value** |  |  |  |  |
| --- | --- | --- | --- | --- | --- | --- | --- |
| Systolic BP, mmHg | 150±20 | 114±6.8 | <0.001 |  |  |  |  |
| Diastolic BP, mmHg | 92±11.1 | 70±6.8 | <0.001 |  |  |  |  |
| Proteinuria, g/24hrs | 2615[365-4170] | N.D |  |  |  |  |  |
| Platelet count, x10^9^/L | 157±64 | 236±38 | 0.005 |  |  |  |  |
| Lactate dehydrogenase | 377.3[238-342] | 165[135-183] | 0.091 |  |  |  |  |
| Gestational age at delivery, in weeks + days † | 29+4±3+4 | 32±3+2 | 0.086 |  |  |  |  |
| Birth weight (grams) | 1211±655 | 3612±618 | <0.001 |  |  |  |  |
| Syndecan -1 (ng/ml) | 461±355 | 809±574 | 0.080 |  |  |  |  |
| Numbers represent mean ± standard deviation, median with [interquartile range], or number of subjects with percentages. †Data missing for 3 women with pre-eclampsia. Between group differences were assessed by t-test for parametric and Mann–Whitney U test for non-parametric distributions. *P*-values were considered to indicate a significant difference if *p*<0.05. | | | | |  |  |  |
